# Combination of Lymph Node Embolization and Musculocutaneous Flap Operation for Managing Groin Lymphorrhea

**DOI:** 10.3400/avd.cr.21-00036

**Published:** 2021-09-25

**Authors:** Pham-Thi Viet Dung, Nguyen Ngoc Cuong, Thai Duy Quang, Pham Hong Canh, Le Tuan Linh, Nguyen Minh Duc

**Affiliations:** 1 Department of Plastic and Reconstructive Surgery, Hanoi Medical University, Ha Noi, Vietnam; 2 Department of Radiology, Hanoi Medical University Hospital, Ha Noi, Vietnam; 3 Department of Radiology, Hanoi Medical University, Ha Noi, Vietnam; 4 Department of Radiology, Pham Ngoc Thach University of Medicine, Ho Chi Minh City, Vietnam; 5 Department of Radiology, Children’s Hospital 2, Ho Chi Minh City, Vietnam

**Keywords:** lymphorrhea, lymph node embolization, myocutaneous flap

## Abstract

Lymphorrhea complications are common following femoral exposure for endovascular procedures. In patients unresponsive to either non-operative or operative therapy, treatment can be complicated. A 86-year-old male patient experienced lymphorrhea after stent graft to treat an abdominal aortic aneurysm, and five operative debridement attempts failed. Intranodal lymphangiography revealed leakage points from two lymph nodes directly into the wound, which were resolved by lymph node embolization using glue. Because the wound was large, a pedicled anterolateral thigh flap (ALT) operation was indicated. Percutaneous lymph node embolization combined with ALT operation may be effective for patients with large wounds and high-flow lymphatic leaks.

## Introduction

Lymphatic leakage is a common complication following vascular surgery in the groin area. Although various treatment options exist for lymphorrhea, the first choice is typically conservative treatment, in which the wound is aspirated using negative pressure, which requires the patient to be hospitalized for several weeks.^1)^ If conservative treatment fails, operative therapy should be considered. Surgical treatment for lymphatic leakage includes lymphatic ligation, removal of debris, and vascularized muscle flaps.^2,3)^ Recently, intranodal lymphatic embolization has been reported to be an effective treatment option.^4)^ Intranodal lymphangiography can be used to identify the leakage point, which cannot be visualized during an open operation. In this article, we illustrated a complicated case of groin lymphorrhea, which was successfully treated through a combination of lymph node embolization and vascularized muscle flaps.

## Case Report

**Figure 1:**
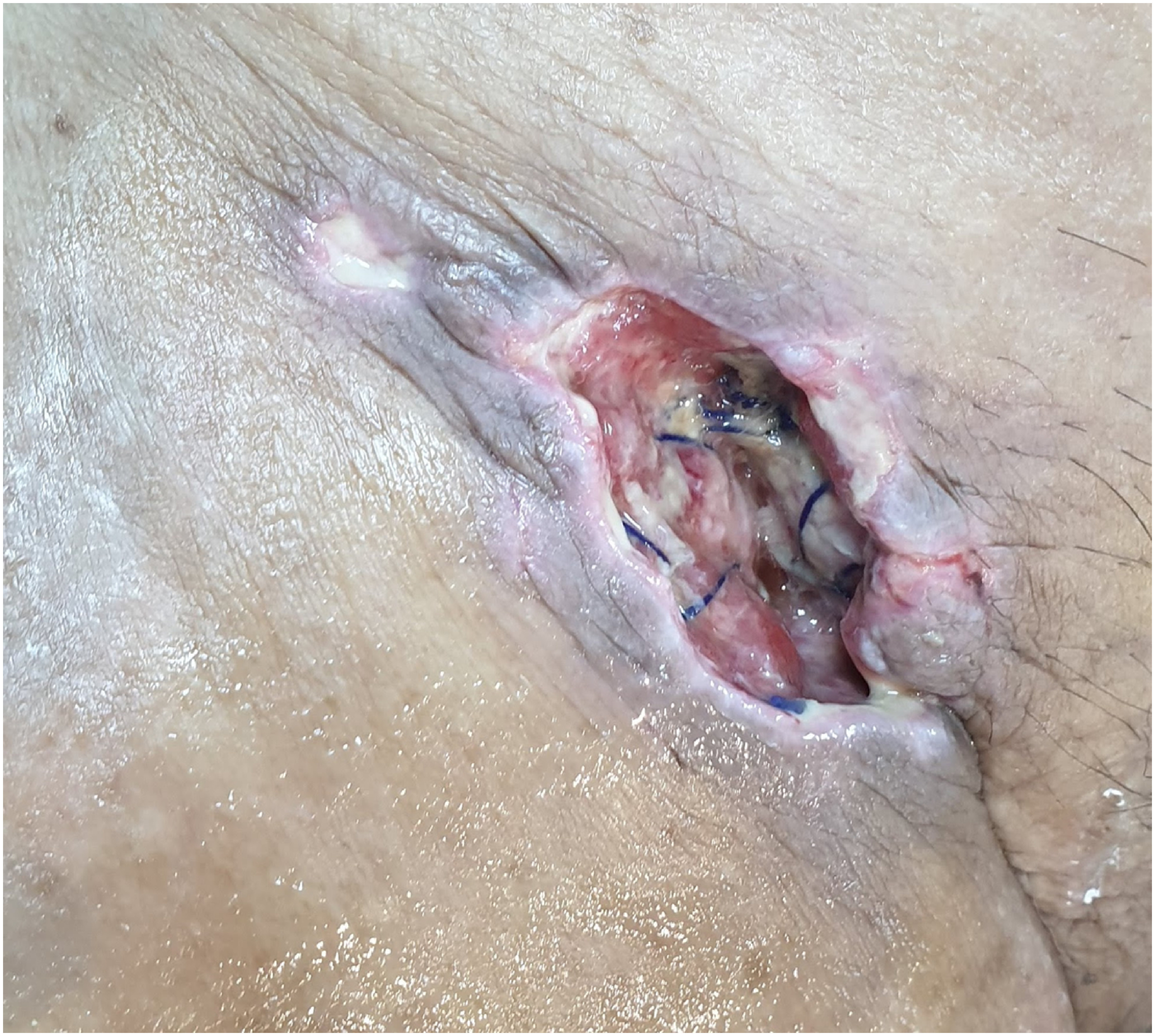
Fig. 1 The open wound in the patient’s right groin upon arrival. Clear fluid continuously welled up from the bottom of the wound.

An 86-year-old man was referred to our institute with groin wound lymphorrhea after repeated, unsuccessful open surgeries. He was initially treated for an abdominal aortic aneurysm with an endovascular stent graft. The procedure was performed through a small incision at the right groin. Unfortunately, after the intervention, the wound failed to heal due to lymphorrhea. The lymphatic leakage volume measured over 300 mL/day through negative pressure drainage. Because of the failure of conservative treatment, the patient had undergone five separate debridement and direct closure operations in the previous hospital, which all failed to heal the wound. The patient was referred to our hospital 8 weeks after the first surgery. The groin defect was approximately 3 cm×5 cm (**Fig. 1**), with continuous lymph leakage, requiring the dressing to be changed approximately seven times per day. A multidisciplinary team of plastic surgeons and interventional radiologists was consulted, and we decided to perform an intranodal lymphangiography to identify the leakage point followed by a pedicled anterolateral thigh flap (ALT) operation.

**Figure 2:**
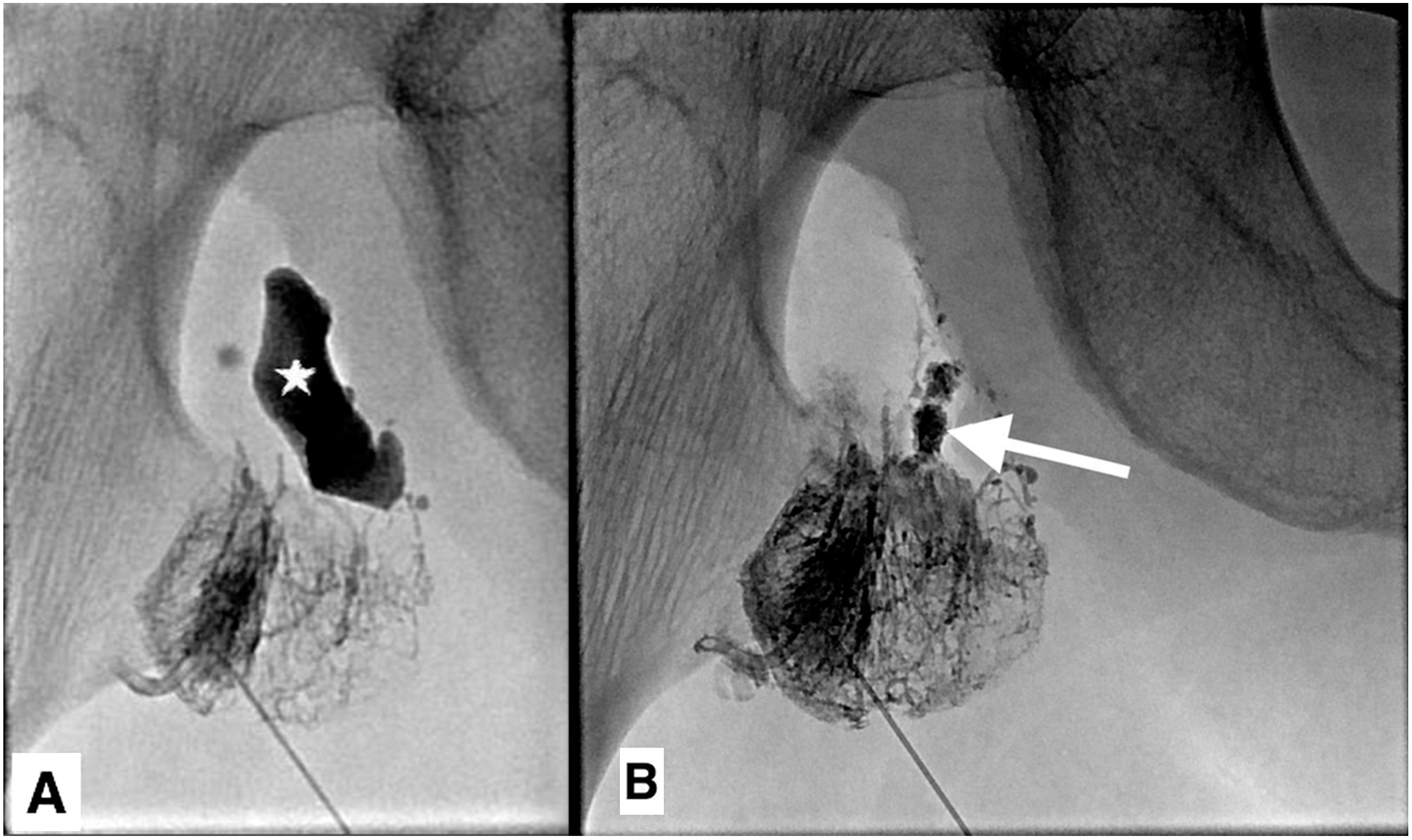
Fig. 2 Lymphatic leak was found and identified using intranodal lymphangiography. (**A**) The contrast agent leaked from the lymph node directly into the wound (star). (**B**) After embolizing the lymph node with NBCA, the leakage point was filled with glue (arrow).

**Figure 3:**
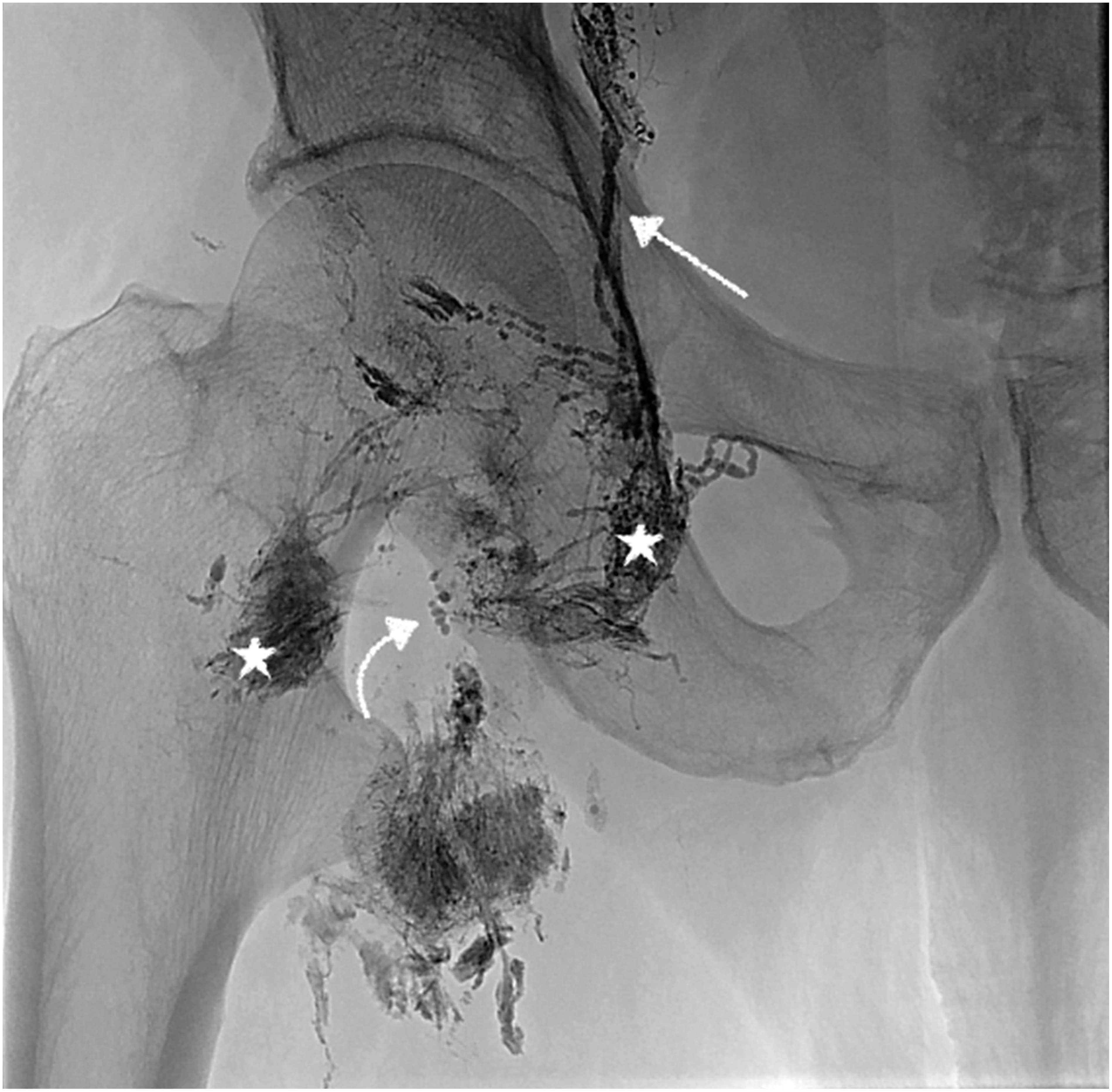
Fig. 3 During the second lymphangiography session, all lymph nodes surrounding the wound were punctured and injected with contrast material (stars). We identified the continuous contrast material extravasation from a lymph node located medially (curved arrow). This lymph node was embolized with NBCA. The normal branches of the lymphatic vessels remained unaffected (straight arrow).

First, the patient underwent intranodal lymphangiography to identify the lymphatic leakage point. The largest lymph node in the groin area, located under the wound, was punctured under ultrasound guidance, and contrast material (Lipiodol, Guerbet, France) was injected into the lymph node. Lymphangiography revealed contrast material extravasation from the lymph node into the wound (**Fig. 2**). An intranodal embolization was performed using N-butyl cyanoacrylate (NBCA) glue. A total volume of 2 mL NBCA diluted with Lipiodol (at a ratio of 1 : 5) was injected into the lymph node. Following the first procedure, although the lymphatic leak volume significantly reduced, the leakage was not completely controlled. A second lymphangiography was performed. During this procedure, all lymph nodes surrounding the wound were punctured (three lymph nodes in total) and injected with contrast material (Lipiodol, Guerbet, France). We identified another leakage point from a lymph node located above the wound (**Fig. 3**). This lymph node was embolized with 0.5-mL NBCA using the same dilution conditions as those of the previous embolization. Following the second embolization, lymphorrhea was successfully controlled.

**Figure 4:**
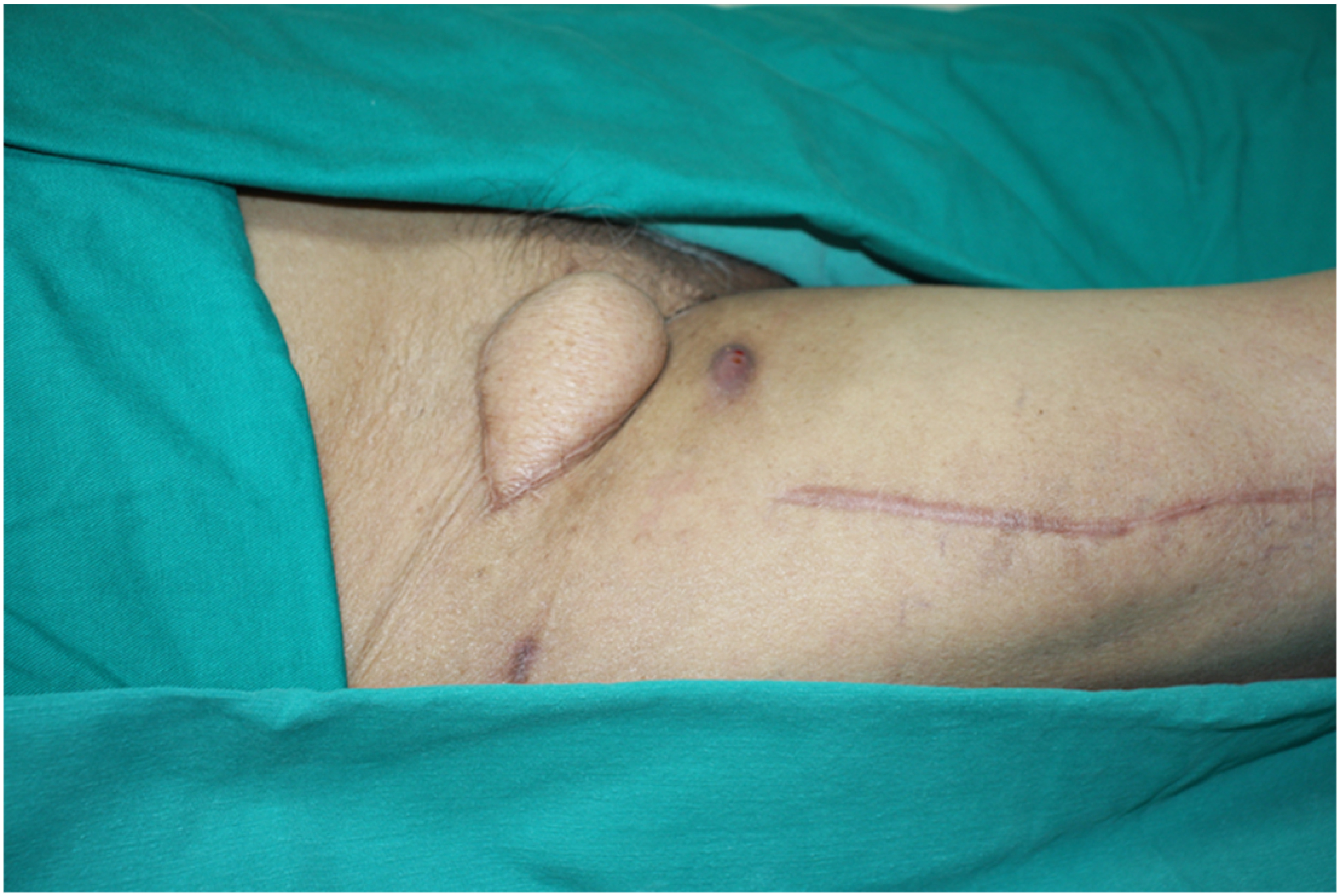
Fig. 4 The wound was healed at reexamination after 6 months of treatment.

Five days after the lymphatic intervention, reconstruction surgery was performed. The chronic wound was widely debrided and meticulously coagulated. After debridement, the defect was large and deep, resulting in the exposure of the femoral artery and veins. A 6 cm×10 cm ALT flap was designed on the basis of a perforator that was identified using a handheld Doppler. The flap was dissected in the subfascial plane, but we included a small portion of the vastus lateralis around the perforator. The dissection proceeded along the descending branch of the lateral circumflex femoral artery until it reached the original branch. The pedicle was distally ligated, and the flap was free to move to the defect under a tunnel. The donor site was closed directly. Negative pressure drainage was placed under the flap and removed after 7 days. The flap uneventfully healed. At reexamination after 6 months, no complication was observed related to either lymph node embolization, with no signs of lymphedema or local pain (**Fig. 4**).

## Discussion

Groin lymphorrhea is an uncommon but well-known complication that can occur following the dissection of the inguinal region for femoral entry during endovascular procedures. The incidence has been reported at approximately 2% of all groin wounds after arterial reconstruction procedures.^5)^ Lymphatic leakage may lead to an unhealed wound, local infection, systemic sepsis, and eventually the loss of limb or death. Traditionally, lymphorrhea is initially managed through non-surgical methods such as compression dressing and negative pressure wound therapy.^1)^ Surgical approaches, including lymphatic ligation, lymphaticovenous anastomosis, and vascularized muscle flaps, are typically only indicated when non-surgical methods fail to resolve the problem.^2,6)^ Lymphatic embolization has been described as a feasible and effective method for managing lymphatic leaks.^7,8)^ In 2018, Smolock et al. reported a series of 10 patients who underwent intranodal embolization for groin lymphorrhea, which resulted in a 100% technical success rate and an 80% clinical success rate.^4)^ Intranodal embolization represents a simple, safe, and effective technique for identifying and treating lymphatic leakage. Moreover, lymphatic embolization requires a shorter procedure and can rapidly resolve the symptoms associated with lymphatic leaks. Typically, lymphatic fluid leaks from lymphatic vessels located under the wound due to the upward flow of lymphatic circulation. In our patient, a second lesion was identified in a lymphatic vessel superior to the wound, which may have been caused by the previously repeated operation. We considered the possibility that all of the lymph nodes surrounding the wound may have been punctured and performed lymphangiography in all proximal lymph nodes to avoid overlooking any lymphatic lesions.

Vascularized muscle flaps, such as sartorius flaps, gracilis flaps, and rectus femoris flaps, have been considered to serve as important methods for groin lymphorrhea treatment, especially in cases of conservative treatment failure.^3)^ However, harvesting a major muscle is always associated with risks, including abdominal herniation and limb function impairment. Occasionally, a bulky flap with a skin graft can lead to an unsatisfactory result. Currently, perforator flaps, especially ALT flaps, represent alternative options for groin reconstruction with several advantages, including reduced donor site morbidity, decreased postoperative pain, and shorter hospitalization stays.^9,10)^ Additionally, either the pedicled ALT flap can provide a large skin flap or it can be harvested as a composite or chimeric myocutaneous flap. In our case, the pedicled ALT flap was elevated using a small portion of the vastus lateralis, which was used to fill the deep and ischemic defect. The inclusion of this muscle portion is thought to control regional infections and may be able to resolve small lymphatic leaks that are difficult to identify using lymphangiography.^2,11)^

In patients with recalcitrant and high-volume lymphatic leakage, the most effective methods have been debated. However, a combination of methods is considered more effective than using any one method alone.^11)^ To our knowledge, no previous description has reported the use of combined techniques to treat a recalcitrant case of lymphorrhea. Further research remains necessary, but we believe that this combination represents a simple and effective method that can definitively solve lymphatic leakage and reduce hospitalization stay.

## Conclusion

Intranodal lymphangiography and lymph node embolization using glue may represent an effective option for postoperative groin lymphorrhea. In patients with large wounds, ALT should be indicated to close the skin.

## Additional Note

The patient has provided written permission to publish these features of his case, and his identity has been protected.
